# Association between elevated iron status and the risk of preeclampsia: A systematic review and meta-analysis

**DOI:** 10.5937/jomb0-64058

**Published:** 2026-05-22

**Authors:** Changhong Li, Xiaohui Xu, Min Qian

**Affiliations:** 1 Hainan Women and Children's Medical Center, Department of Obstetrics, Haikou, China; 2 General Hospital of Eastern Theater Command, PLA, Department of Obstetrics and Gynecology, Nanjing, China

**Keywords:** hepcidin, iron, meta-analysis, preeclampsia, hepcidin, gvožđe, meta-analiza, preeklampsija

## Abstract

**Background:**

This review aims to conduct a comprehensive and systematic report of the currently published information on the association between elevated hepcidin levels and the risk of preeclampsia by meta-analysis of published prospective case-control studies or cross-sectional studies combined with follow-up to provide a comprehensive and reliable basis for early intervention and clinical decision making in the disease.

**Methods:**

We searched five databases such as Pubmed, Web of Science, Cochrane, China National Knowledge Infrastructure, and WAN FANG DATA to retrieve articles related to the relationship between elevated levels of hepcidin and the risk of preeclampsia up to February 2025. We calculated the standardized mean deviation (SMD) and 95% confidence interval (95% CI) for comparison using a random-effects model.

## Abstract (sr)

**Uvod:**

Cilj ovog pregleda je da sprovede sveobuhvatan i sistematski izveštaj' o trenutno obj'avlj'enim informacij'ama o povezanosti između povisenih nivoa hepcidina i rizika od preeklampsije putem meta-analiza objavljenih prospektivnih studij'a slučaj'-kontrola ili poprečnih studij'a u kombinaciji sa pracenjem, kako bi se obezbedila sveobuhvatna i pouzdana osnova za ranu intervencju i kliničko donošenj'e odluka kod ove bolesti.

**Metode:**

Pretražili smo pet baza podataka kao sto su Pubmed, Web of Science, Cochrane, China National Knowledge Infrastructure i WAN FANG DATA kako bismo pronašli članke koji se odnose na vezu između povisenih nivoa hepcidina i rizika od preeklampsije do februara 2025. godine. Izračunali smo standardizovanu srednju devijaciju (SMD) i 95% interval poverenja (95% CI) za poređenje koristeci model slučajnih efekata.

**Rezultati:**

Ukupno 8 članaka je uključeno u ovaj pregled. Rezultati su pokazali da su kod trudnica sa preeklampsijom primeceni visi nivoi serumskog hepcidina i serumskog gvožđa i niži nivoi serumskog transferina u poređenj'u sa normotenzivnim trudnicama (serumski hepcidin: SMD = 1,08, 95%CI: 0,03, 2,14; serumsko gvožđe: SMD = 0,63, 95%CI: 0,41, 0,86; serumski transferin: SMD = -0,50, 95%CI: -0,73, -0,27).

**Zaključak:**

Slično prethodnom pregledu, ovaj sistematski pregled i meta-analiza pokazali su da visoki nivoi gvožđa mogu biti povezani sa vecim rizikom od preeklampsije. Pored toga, ova meta-analiza je sumirala dva mehanizma za neuspeh hepcidina da smanje nivoe gvožđa u serumu kod trudnica sa preeklampsijom u kasnoj trudnoci. I ova meta-analiza bi vodila dalja klinička istraživanja.

## Introduction

Pre-eclampsia (PE) is a pregnancy-specific syndrome affecting approximately 3%-8% of pregnancies and remains a leading cause of maternal and fetal morbidity and mortality [1]. PE typically develops after 20 weeks of gestation and is characterized by new-onset hypertension (systolic blood pressure ≥ 140 mmHg and/or diastolic blood pressure ≥ 90 mmHg) accompanied by proteinuria (≥ 300 mg/24 h) [2][3][4]. Although the etiology and pathogenesis of PE are not fully understood, endothelial injury and systemic inflammation are considered central features and are closely linked to disturbances in iron homeostasis. Iron is essential for numerous physiological processes, yet excess free iron is potentially toxic because it can catalyze free-radical generation. Under dysregulated iron homeostasis, free iron can drive reactive oxygen species production via the Fenton reaction, generating highly reactive hydroxyl radicals and other oxidants. These species trigger free-radical chain reactions, resulting in lipid peroxidation and DNA damage, which are associated with apoptosis, necrosis, and increased autophagy [5][6][7].

At present, serum iron and ferritin have been reported their changes during pregnancy closely related to the incidence of preeclampsia [8][9][10]. One of the significant roles of hepcidin is maintaining iron homeostasis, where they reduce body iron levels by inhibiting intestinal iron uptake and iron release from macrophages and senescent erythrocytes [11]. Besides, it is believed that hepcidin and iron homeostasis regulate each other. Pigeon C, et al. [12] found that either dietary iron overload or hepatic iron overload led to overexpression of hepatic hepcidin mRNA in mice, whereas iron deficiency reduced its expression. In other words, iron deficiency will lower the expression of hepcidin, and thereby increase the levels of body iron. On the contrary, iron overload will increase the expression of hepcidin to decrease the body's iron level by lowering the absorption of iron [11].

Elevated hepcidin levels during pregnancy may reduce circulating free iron and limit iron-mediated cytotoxicity by restricting iron export through transporters (e.g., ferroportin) and promoting iron sequestration in ferritin. However, as preeclampsia progresses—particularly in severe cases—this regulatory balance may become disrupted, potentially harming both the mother and fetus. Accordingly, hepcidin has been proposed as a biomarker to help evaluate fetal outcomes and support timely intervention [11]. In recent years, evidence regarding hepcidin as a diagnostic marker and indicator of preeclampsia progression has been inconsistent. For instance, Cardaropoli et al. [13] reported no significant difference in hepcidin levels between women with preeclampsia and normotensive controls during the second half of pregnanc. In contrast, Nila et al. [14] observed significantly different hepcidin levels between the two groups and suggested that hepcidin could serve as a diagnostic predictor of fetal outcome in preeclampsi. Therefore, this systematic review and meta-analysis aims to synthesize the available evidence on the association between elevated hepcidin levels and the risk of developing preeclampsia. We will meta-analyze published prospective case-control studies and cross-sectional studies with follow-up to provide a comprehensive and reliable evidence base to inform early intervention and clinical decision-making.

## Materials and methods

This systematic review followed the methodology outlined in the Cochrane Handbook for Systematic Reviews of Interventions Version 6.0 [15]. In addition, we described it according to the PRISMA-P (Preferred Reporting Items for Systematic Reviews and Meta-Analyses Protocols) statement [16].

### Search strategy

We searched five electronic databases, Pubmed, Web of Science, Cochrane, China National Knowledge Infrastructure, and WAN FANG DATA. Furthermore, we systematically searched for articles related to the association between elevated hepcidin levels and the risk of preeclampsia development up to February 2025. The search was conducted for the following subject terms: «preeclampsia«, «pregnancy-induced hypertension«, «hepcidin«, «iron status«, «ferritin«, «transferrin«, «iron deficiency«, and «iron overload«. The detailed search strategies in the different electronic databases were listed in Table 1. In addition, we meticulously searched the references of articles that were systematically searched and included in the meta-analysis to not leave behind any article that reported valid analysis data and provide comprehensive coverage of articles related to the association of elevated hepcidin levels with the risk of developing preeclampsia.

**Table 1:** The detail of search strategy in Meta-analysis.

**Pubmed**
((preeclampsia) OR (preeclampsia[MeSH Terms]) OR (pre-eclampsia) OR
(pre-eclampsia[MeSH Terms]) OR (hypertension, pregnancy induced) OR (hypertension, pregnancy induced[MeSH Terms])) AND ((hepcidin) OR (hepcidin[MeSH Terms]) OR (iron status) OR (ferritin) OR (ferritin [MeSH Terms]) OR (transferrin) OR (transferrin [MeSH Terms]) OR (iron deficiencies) OR (iron deficiencies[MeSH Terms]) OR (overload, iron[MeSH Terms]) OR (overload, iron))
**Web of science**
(TS=(preeclampsia) OR TS=(pre-eclampsia) OR TS=(pregnancy induced hypertension)) AND
(TS=(hepcidin) OR TS=(iron status) OR TS=(ferritin) OR TS=(transferrin) OR TS=(iron deficiencies) OR TS=(iron overload))
**Cochrane**
((preeclampsia) OR (pre-eclampsia) OR (pregnancy induced hypertension)) AND ((hepcidin)
OR (iron status) OR (ferritin) OR (transferrin) OR (iron deficiencies) OR (iron overload)).
Searching was conducted in TITLE, ABSTRACT and KEYWORDS.
**China National Knowledge Infrastructure**
Subject terms: «preeclampsia«, «pre-eclampsia«, «pregnancy-induced hypertension«, «hepcidin«, «iron status«, «ferritin«, «transferrin«, «iron deficiency«, and «iron overload« were chosen to search in TITLE, ABSTRACT and KEYWORDS in the same Boolean logic of pubmed.
**WAN FANG DATA**
Subject terms: «preeclampsia«, «pre-eclampsia«, «pregnancy-induced hypertension«, «hepcidin«, «iron status«, «ferritin«, «transferrin«, «iron deficiency«, and «iron overload« were chosen to search in TITLE, ABSTRACT and KEYWORDS in the same Boolean logic of pubmed.

### Inclusion and exclusion criteria

Two researchers independently evaluated the titles and abstracts of articles retrieved from the database system to determine whether they met the inclusion or exclusion criteria for inclusion in the study. In case of disagreement between the two researchers, a third researcher would be consulted, and the third researcher would make the final decision on the selection of articles based on the opinions of both researchers. The inclusion criteria were (1) the study should be a case-control study or a cross-sectional study with multiple follow-ups. (2) study population was: 1) primary: pregnant women and divided into study and control groups according to the presence or absence of preeclampsia; 2) secondary: infants and mothers and divided into study and control groups according to the presence or absence of adverse birth outcomes (adverse outcomes defined as preterm birth before 34 weeks of gestation, placental abruption before delivery, and neonatal stay in the neonatal intensive care unit for more than 24 hours). (3) The study recorded the age, gestational age, iron status, and levels of iron-regulating factors. Exclusion criteria (any of the following conditions were excluded) were: (1) the study was a duplicate publication of the same study results (2) the full text was unavailable or extracting the required data from the full text is impossible; (3) the reporting of data was incomplete and relevant data through credible sources were unavailable.

### Data extraction and quality assessment

Two investigators independently extracted data from each eligible study using a standardized data-extraction form. The extracted information included the study title; first author and year of publication; eligibility/selection criteria; participant grouping and sample size; maternal age and gestational age; diagnostic criteria for preeclampsia; indices of iron status; hepcidin levels; and study design-related information (primarily the study protocol and quality-control procedures). After extraction, a third investigator cross-checked the two datasets and resolved any inconsistencies.

Studies that met the inclusion criteria had their quality assessed independently by two authors using the Newcastle-Ottawa Scale (NOS) case-control study component [17]. The assessment was made of (1) the appropriateness of the selection of cases and controls: 1) the definition of cases and controls; 2) the sources of case and control selection; (2) the comparability of cases and controls; and (3) the appropriateness of exposure determination. Studies were rated as high quality if they scored 6-9; moderate quality if they scored 4 or 5, and poor quality if they scored three or lower. If there were disagreements between the two researchers, a third researcher participated in the discussion and made the final determination of the study's score based on the opinions of the previous two researchers.

### Statistical analysis

Results were merged across studies with STATA version 15.1 (Stata Corp MF), College Station, TX, USA) [18][19]. Considering the essential characteristics of the study population, such as maternal age, gestational age, BMI, iron supplementation status, and different study areas between studies, there was no doubt a non-negligible clinical heterogeneity in this meta-analysis. Therefore, we used a random-effects model to combine study indicators to discuss the relationship between elevated hepcidin levels and the risk of developing preeclampsia. In addition, discussing the sources of heterogeneity in the between-study effect indicators helped improve our understanding of the association between elevated hepcidin levels and the risk of developing preeclampsia. For continuous variables, we used the standardized mean difference (SMD) and its 95% confidence interval (CI) to compare whether the study indicator was a critical factor in the increased risk of developing preeclampsia in patients with preeclampsia. In addition, the method for calculating the combined standard deviation (SD) of the studies was referred to the Cochrane Handbook [15]. Q test and I^2^ statistics were used to assess the heterogeneity of the studies. I^2^ values of 0%-39%, 40%-59%, and 60%-90% were considered as low, medium, and high heterogeneity between studies, respectively [15]. When evaluating hepcidin and indicators of iron status in pregnant mothers with preeclampsia versus normal pregnancy, we would display the results using forest plots. We used Egger's test to assess the publication bias of the results and Duval and Tweedie's trim and fill test to assess the sensitivity of the results [20][21]. We would give exact P values unless P < 0.001. P < 0.05 might be considered statistically significant except for Egger's test, where P < 0.10 was considered statistically significant.

## Results

### Literature search, study characteristics and quality assessment

**Figure 1:**
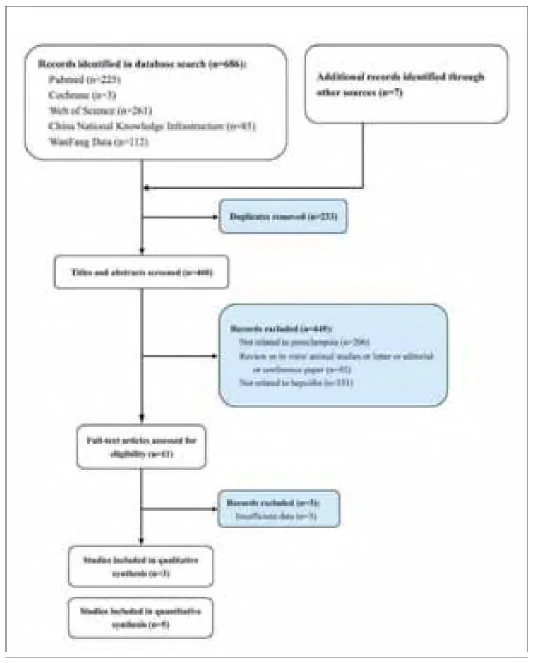
Study selection flowchart, systematic review and meta-analysis of association of elevated hepcidin levels with the risk of preeclampsia.

686 and 7 articles were obtained through the database system search and manual search of references, respectively. First, the comparison revealed the existence of 233 duplicate retrieved articles. Second, the titles and abstracts of the retrieved articles were screened after removing the duplicate retrievals. 460 articles that did not meet the inclusion criteria were excluded (not related to preeclampsia n = 206; review or in vitro/animal studies or letter or editorial or conference paper n = 92; not related to hepcidin n = 151). Third, three out of the following 11 articles subjected to full-text assessment were excluded because they did not provide or translate into validly usable data (mainly without hepcidin-related data available in the full text of the article). Finally, three studies were included in the qualitative analysis for the reasons of only got infographic and P value related to hepcidin level in PE patients and normotensive pregnant women, or the study object included PE patients combine with other adverse pregnancy outcome patients. While we got no additional assistance from the authors after we contacted them. Five articles were included in the quantitative analysis (Figure 1). A total of 184 pregnant women with preeclampsia and 219 pregnant women with normal pregnancies were included in this review for meta-analysis. The primary characteristics of the four studies included in the meta-analysis are shown in Table 2 [14][22][23][24][25].

**Table 2:** Baseline characteristics of included studies for meta-analysis. Note: Forty diagnosed cases of preeclampsia and forty normotensive women with uncomplicated pregnancies both between the gestational ages of 34 ± 4 weeks belonging to the age group 18-40 years were included in the study. Abbreviation: PE = preeclampsia; Ctrl = control; y = year; wk = week; NOS = Newcastle-Ottawa Scale.

First<br>author,<br>year	No. of<br>cases(PE /<br>Ctrl)	Maternal age<br>(y, PE / Ctrl)			Gestational age at<br>sampling (wk, PE / Ctrl)				Definition of PE	Study design	NOS
Nila SG,<br>2019	40 / 40	24.85	±	3.90 /	33.00	±	3.40	/	Systolic blood pressure of > 140<br>mm of	Cross-sectional	8
		24.60	±	3.60	33.60	±	3.20		Hg or a diastolic blood pressure of<br>> 90	study with<br>follow	
									mm of Hg at two separate occasions	up	
									minimum 4 h apart with or without<br>24 h		
									urine protein > 300 mg/ day or urine		
									protein/creatinine ratio > 0.3.		
Toldi G,<br>2010	30 / 37	30.00	±	7.00 /	36.50	±	4.00	/	Systolic blood pressure of > 140<br>mm of	Cross-sectional	7
		30.00	±	4.00	36.00	±	2.75		Hg or a diastolic blood pressure of<br>> 90	study with<br>follow	
									mm of Hg at two separate occasions	up	
									minimum 4 h apart with or without<br>24 h		
									urine protein > 300 mg/ day or urine		
									protein/creatinine ratio > 0.3.		
Shaji<br>Geetha N.<br>2020	40 / 40	Note			34.00	±	4.00	/	Systolic blood pressure of > 140<br>mm of		7
					34.00	±	4.00		Hg or a diastolic blood pressure of<br>> 90		
									mm of Hg at two separate occasions	up	
									minimum 4 h apart with or without<br>24 h		
									urine protein > 300 mg/ day or urine		
									protein/creatinine ratio > 0.3.		
Brunacci F,<br>2018	18 / 18	22.00	±	8.50 /	31.50	±	6.00	/	Systolic blood pressure of > 140<br>mm of	Cross-sectional	6
		27.00	±	6.00	31.50	±	3.30		Hg or a diastolic blood pressure of<br>> 90	study with<br>follow	
									mm of Hg at two separate occasions	up	
									minimum 4 h apart with or without<br>24 h		
									urine protein > 300 mg/ day or urine		
									protein/creatinine ratio > 0.3.		
Zhang LX,<br>2021	66 / 84	31.00	±	2.83 /	33.00	±	2.00	/	Systolic blood pressure of > 140<br>mm of	Prospective	9
		30.00	±	3.12	33.00	±	2.00		Hg or a diastolic blood pressure of<br>> 90	observational	
									mm of Hg at two separate occasions	case-control<br>study	
									minimum 4 h apart with or without<br>24 h		
									urine protein > 300 mg/ day or urine		
									protein/creatinine ratio > 0.3.		

The NOS quality assessment scale scored the five included studies between 6 and 9, with an appropriate selection of cases and controls, reliable study metric measures, and overall study quality considered high. Table 2 shows the results of their scores. And the detail of assessment items was shown in Table 3. It is noteworthy that before study initiation, most studies excluded pregnant women with the following conditions: pregnant women with gestational diabetes, anemia during pregnancy (Hb < 11 g/dL), severe pregnancy vomiting, thyroid disease, preeclampsia combined with chronic hypertension, pregnant women with chronic hypertension, current pregnancy infection, renal disease, significant congenital anomalies, autoimmune disease, obese subjects, and smokers. The studies thus did not have significant missing data that would have seriously compromised test validity but a limited extrapolation of the findings. In summary, the overall quality assessment of the studies included in this meta-analysis is of good quality with highly reliable results.

**Table 3:** The detail of NOS quality assessment of five included papers.

	Nila SG,<br>2019	Toldi G,<br>2010	Shaji<br>Geetha N.<br>2020	Brunacci F<br>2018	Zhang LX,<br>2021
Is the case definition adequate?	1	1	1	1	1
Representativeness of the cases	1	1	1	1	1
Selection of controls	0	0	0	0	1
Definition of controls	1	1	1	1	1
Comparability of cases and controls on the<br>basis of the design or analysis	2	1	1	0	2
Ascertainment of exposure	1	1	1	1	1
Same method of ascertainment for cases and<br>controls	1	1	1	1	1
Non-Response Rate	1	1	1	1	1
**Total score**	**8**	**7**	**7**	**6**	**9**

### Indicators

### Comparison between pregnant woman with preeclampsia and gestational woman with normotension

**Figure 2:**
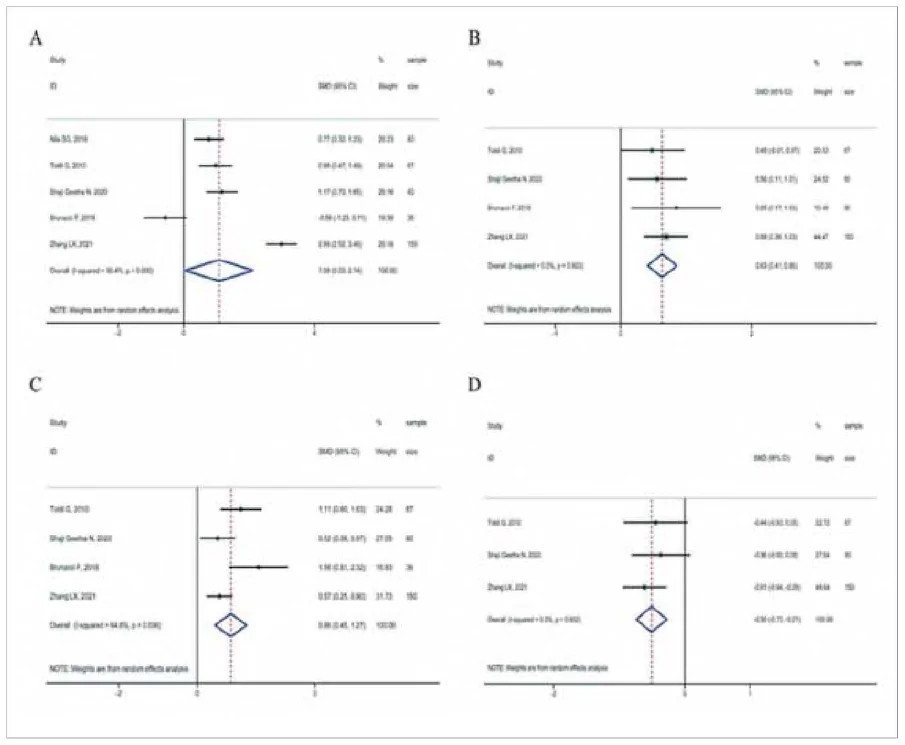
Forest plot of pregnant woman with preeclampsia and gestational woman with normotension: (A) hepcidin; (B) plasma iron; (C) plasma ferritin; (D) plasma transferrin.

Hepcidin. The meta-analysis of five studies on plasma hepcidin levels in 184 pregnant women with preeclampsia compared with 219 normotensive pregnant women showed higher plasma hepcidin levels in pregnant women with preeclampsia compared with normotensive pregnant women, and the difference was statistically significant (SMD = 1.08, 95%CI: 0.03, 2.14; P = 0.044; Figure 2A) [14][22][23][24][25].

However, it is noteworthy that only study Brunacci F 2018 out of five studies showed lower plasma hepcidin levels in women with preeclampsia than in women with normal blood pressure, and the difference was not statistically significant (SMD = -0.56, 95%CI: -1.23, 0.11; P = 0.100; Figure 2A) [24]. Additionally, the plasma levels of hepcidin were higher in women with preeclampsia in the remaining four studies, and the differences were statistically significant. This point deserves further in-depth discussion [14][22][23][25].

Iron status. 4 studies compared plasma iron levels in 144 preeclamptic versus 179 normotensive pregnant women, and the meta-analysis results showed higher blood Iron levels in pregnant women with preeclampsia, with a statistically significant difference (SMD = 0.63, 95%CI: 0.41, 0.86; P < 0.001; Figure 2B) [22][23][24][25]. In addition, four studies reported plasma ferritin concentrations in 144 preeclamptic versus 179 normotensive pregnant women. The combined results of the four studies showed higher plasma ferritin concentrations in preeclamptic pregnant women, and the difference was statistically significant (SMD = 0.86, 95%CI: 0.45, 1.27; P < 0.001; Figure 2C) [22][23][24][25]. 3 studies provided mean plasma transferrin levels in 126 preeclamptic versus 161 normotensive pregnant women, and the meta-analysis showed lower plasma transferrin concentrations in preeclamptic pregnant women (SMD = -0.50, 95%CI: -0.73, -0.27; P < 0.001; Figure 2D) [22][23][25].

### Publication bias assessment and sensitivity analysis

Egger's test was used to analyze the publication bias of hepcidin, plasma iron, and plasma ferritin indicators. It suggested no significant publication bias for any of the above indicators. Besides, Duval and Tweedie's trim and fill sensitivity test suggested that these indicators were stable after trimming and filling. None of them appeared to essential change. Therefore, the guiding significance of these indicators was clear (Table 4).

**Table 4:** Evaluation of publication bias and sensitivity analysis.

Index	Egger's regression		Duval and Tweedie's trim and fill		
	Intercept	*p*	Original effect size<br>(95% CI)	Studies<br>trimmed	
Hepcidin	-20.124	0.275	1.08 (0.03, 2.14)	0	1.08 (0.03, 2.14)
Plasma iron	0.158	0.922	0.63 (0.41, 0.86)	0	0.63 (0.41, 0.86)
Plasma ferritin	4.646	0.117	0.86 (0.45, 1.27)	1	0.71 (0.26, 1.15)

## Discussion

First of all, it should be emphasized that this meta-analysis focused on the relationship between elevated hepcidin levels and the risk of preeclampsia. Meantime, the data on iron status in the included studies were recorded and compared to facilitate discussion and analysis of the role of hepcidin concerning iron homeostasis in pregnant women with preeclampsia. Due to the small number of included meta-analysis studies, the meta-analysis results on iron status in preeclamptic women in this review were not representative. However, the results of this meta-analysis are consistent with the findings of Song QY [26], a previous systematic review that included 24 studies focused on high serum iron levels and increased risk of preeclampsia disease (plasma iron: SMD = 1.27, 95%CI: 0.76, 1.78; I^2^ = 96%). In addition, another study by Lewandowska M [27] showed that in early pregnancy (10-14 weeks) in preeclamptic women, serum iron levels showed a trend to be higher than in normotensive women and then gradually became lower than in normotensive women. After examining the key characteristics of the studies included in the systematic review by Song QY [26], we speculate that the absence of gestational age-stratified analyses across studies may have contributed to the substantial heterogeneity observed. In summary, elevated serum iron levels appear to be associated with an increased risk of preeclampsia; however, future studies should provide detailed, trimester-specific assessments (early, mid, and late pregnancy) to better clarify temporal patterns.

In this study, we confirmed that high plasma ferritin levels were associated with a higher risk of preeclamptic disease. First, we noticed all included in quantitative meta-analysis articles were shown higher levels of serum ferritin in preeclampsia patients than in normotensive pregnant women. We noticed other studies that focused on the relationship between serum ferritin and preeclampsia. The study by Yao BL [28] indicated that plasma ferritin was slightly higher in pregnant women with simple preeclampsia compared to those with normal blood pressure, both in early, mid, and late pregnancy, but the difference was not statistically significant (P > 0.05). In contrast, the plasma ferritin concentration in pregnant women with gestational diabetes combined with preeclampsia was higher in mid-and-late pregnancy than in those with normal blood pressure, and the difference was statistically significant (P < 0.05) [28]. In summary, the study indicates that elevated serum ferritin might not be a cause of preeclampsia but rather an essential predictor of disease severity, especially when serum ferritin levels are greater than or equal to 32.3 mg/L in mid-pregnancy or serum ferritin levels reached 41.6 mg/L in late pregnancy, suggesting more severe disease [28]. Therefore, timely monitoring of serum ferritin during pregnancy may not only guide appropriate iron supplementation, but also help inform clinical decision-making.

The basic characteristics of the three articles for qualitative analysis are shown in Table 5. Besides, raw data of included studies for meta-analysis were listed in Table 6. The study by Simavli S, [29] used adverse pregnancy outcomes (APO including 18 pregnant women with preeclampsia and five pregnant women with gestational hypertension) as the basis for grouping. The results showed that the APO group exhibited higher hepcidin levels in early, mid, and late pregnancy than the control group, but the difference was not statistically significant. In contrast, the APO group showed higher serum iron levels in early and late pregnancy, with statistically significant differences [29]. On the other hand, the prospective case-control study by Cardaropoli S [13] verified that hepcidin levels in women with late pregnancy preeclampsia were already altered in early pregnancy, suggesting that high maternal serum hepcidin levels might be an early marker of preeclampsia. Additionally, the study by Duvan CI [30] used pro-hepcidin concentrations in preeclamptic mothers as an acute phase reactant to investigate its relationship with iron homeostasis to search for peptide hormones that might alter preeclampsia status. Unfortunately, the difference between pro-hepcidin as a precursor of hepcidin in preeclamptic women and normotensive women was not statistically significant, demonstrating no observable acute inflammatory response in preeclamptic women [30].

**Table 5:** Baseline characteristics of included studies for qualitative analysis. Abbreviation: Res = research; Ctrl = control; y = year; wk = week; NOS = Newcastle-Ottawa Scale.

First author,<br>year	No. of<br>cases (Res<br>/ Ctrl)	Maternal age (y,<br>Res / Ctrl)			Gestational age at<br>sampling (wk, Res / Ctrl)				Definition of Res	Study design	NOS
Simavli S,<br>2015	23 / 103	27.60	±	4.10 /	37.00	±	2.00	/	Res: adverse<br>pregnancy<br>outcomes group	Prospective	5
		27.50	±	3.90	39.00	±	3.10		which include 18<br>pregnant women<br>with	cohort study	
									preeclampsia and 5<br>pregnant women		
									with gestational<br>hypertension		
Cardaropoli<br>S, 2018	45 / 60	34.20	±	4.70 /	33.30	±	4.50	/	Res: preeclampsia<br>which systolic blood	Cross-sectional	8
		31.60	±	4.60	33.70	±	5.50		pressure of > 140<br>mm of Hg or a	study with<br>follow	
									diastolic blood<br>pressure of > 90<br>mm of	up	
									Hg at two separate<br>occasions minimum		
									4 h apart with or<br>without 24 h urine		
									protein > 300 mg/<br>day or urine		
									protein/creatinine<br>ratio > 0.3.		
Duvan CI,<br>2015	30 / 30	30.20	±	4.90 /	35.20	±	4.10	/	Res: preeclampsia<br>which systolic blood	Prospective	7
		28.40	±	5.40	39.30	±	1.10		pressure of > 140<br>mm of Hg or a	observational	
									diastolic blood<br>pressure of > 90<br>mm of	case-control<br>study	
									Hg at two separate<br>occasions minimum		
									4 h apart with or<br>without 24 h urine		
									protein > 300 mg/<br>day or urine		
									protein/creatinine<br>ratio > 0.3.		

**Table 6:** Raw data of meta-analysis. Data were shown in median (range) in article Toldi G, 2010, median (inter quartile range) in article Nila SG, 2019, Shaji Geetha N. 2020, Brunacci F, 2018 and Zhang LX, 2021 unless the data of plasma transferrin in article Zhang LX, 2021 was shown in mean (SD).

Author	n1	average1	dispersion1	n2	average2	dispersion2	sample size
Hepcidin							
Nila SG, 2019 (pg/mL)	40	683	595-843	40	558	425-610	80
Toldi G, 2010 (ng/mL)	30	5.68	0.72-9.25	37	3.74	0.73-8.14	67
Shaji Geetha N. 2020 (pg/<br>mL)	40	684	595-684	40	558	425-610	80
Brunacci F, 2018 (ng/mL)	18	46.52	39.92-51.66	18	51.46	47.73-59.81	36
Zhang LX, 2021 (ng/mL)	66	40.71	42.69-33.88	84	25.95	23.99-28.28	150
Plasma iron							
Toldi G, 2010 (mmol/L)	30	19.1	7.1-51.6	37	15	6.8-29.5	67
Shaji Geetha N. 2020 (mg/<br>dL)	40	588	367-972	40	390	248-544	80
Brunacci F, 2018 (mg/dL)	18	161.33	117.2-194.7	18	116.79	82.45-145.74	36
Zhang LX, 2021 (mg/L)	66	10.73	9.25-13.22	84	9.11	7.18-12.04	150
Plasma ferritin							
Toldi G, 2010 (mg/L)	30	34	5-78	37	15	5-69	67
Shaji Geetha N. 2020<br>(mg/L)	40	38	19-74	40	22	12-32	80
Brunacci F, 2018 (ng/mL)	18	72.5	47.25-112.25	18	17	8.75-27.75	36
Zhang LX, 2021 (ng/mL)	66	18.6	11.23-29.15	84	14.34	10.94-19.95	150
Plasma transferrin							
Toldi G, 2010 (mmol/L)	30	4.1	2.8-5.7	37	4.4	3.6-6.2	67
Shaji Geetha N. 2020<br>(mg/dL)	40	617	501-778	40	705	537-910	80
Zhang LX, 2021 (ng/mL)	66	3.38	0.75	84	3.77	0.54	150

Two hypotheses may explain why hepcidin remains elevated in early and late pregnancy and why it may fail to adequately regulate maternal serum iron levels in preeclampsia, thereby offering directions for future research. First, evidence suggests that serum iron levels are higher in women who develop preeclampsia than in normotensive women in early pregnancy (10-14 weeks). However, despite comparable iron intake, serum iron in preeclamptic women appears to decline with advancing gestation and may ultimately fall below levels observed in uncomplicated pregnancies [27]. Meanwhile, the study by Cardaropoli S [13] verified that hepcidin levels in women with preeclampsia were higher in early pregnancy than in women with normal pregnancies. Thus the changes in serum iron levels in women with preeclampsia in the study by Lewandowska M [27] might be plausibly explaine. In addition, we could observe the studies included in the meta-analysis to find that serum iron levels continue to be higher in women with preeclampsia in late pregnancy than in women with normal blood pressure. It was also important to note that except for the study of Brunacci F [24] the SMD of serum hepcidin levels in the other three studies were greater than 0, and the differences were statistically significant. The small sample size in the paper Brunacci F [24], which could cause selection bias might explain its' result got a lower hepcidin level, and with a non-significant statistical difference. Moreover, for the reason that the study by Brunacci F [24], got 19.39% weight in the meta-analysis only including 18 preeclampsia patients in research, it's easy to understand why we cannot observe a distinct elevation of hepcidin in preeclampsia patients in the meta-analysis. Therefore, the study by Brunacci F [24], concluded that serum iron remained high because of reduced serum hepcidin production. Such a conclusion was not tenable in the studies of Toldi G [22], Shaji Geetha N, [23]. Instead, these three studies believed that serum iron levels in preeclamptic women were resistant to the regulation of hepcidin [22][23][25]. Regardless of which of the above hypotheses better explains the increased risk of preeclampsia, the hepcidin-to-iron status ratio may be a useful indicator for predicting preeclampsia risk. This approach was applied by Brunacci F [24] and showed a distinct effect. Overall, after controlling for iron intake in women with preeclampsia and normotensive pregnant women, incorporating a hepcidin/serum iron ratio may help future studies address the existing controversy, clarify why hepcidin fails to regulate maternal serum iron in late pregnancy, and inform clinical management [26][27][28][29][30].

### Limitations

The main limitation of this meta-analysis was that the number of studies on this topic was too small, but a timely summary would help advance further clinical studies. However, more prospective case-control studies with large samples from different regions and prospective cohort studies designed around the possible pathogenesis of preeclampsia need to be kept in focus, which could help clarify the role of hepcidin in the pathogenesis of preeclampsia and thus guide clinical practice [31]. Besides, what role play of hepcidin in the severity of preeclampsia was not addressed in the included studies.

## Conclusion

Similar to the previous review, the results of this systematic review and meta-analysis results indicated that high serum iron levels might be associated with a higher risk for preeclampsia. In addition, this meta-analysis summarized two mechanisms for the failure of hepcidins to reduce serum iron levels in pregnant mothers with preeclampsia in late pregnancy. And this meta-analysis would guide further clinical studies.

## Dodatak

### Funding

This work was supported by the project supported by Hainan Province Clinical Medical Center (QWYH202175).

### Abbreviation

PE = Pre-eclampsia; NOS = Newcastle-Ottawa Scale SMD = Standard Mean Difference CI = Confidence Interval; SD = Standard Deviation; APO = Adverse Pregnancy Outcomes.

### Conflict of interest statement

All the authors declare that they have no conflict of interest in this work.

### Contribution

Changhong Li and Xiaohui Xu contributed equally to this work.
